# Combined effects of drought intensity and heat stress impair physiological performance and recovery capacity in *Populus nigra* L. seedlings

**DOI:** 10.1093/treephys/tpag078

**Published:** 2026-06-11

**Authors:** Tsvetana Masante, Peter Beatrice, Chiara Pezzuto, Alessio Miali, Gabriella Stefania Scippa, Antonio Montagnoli

**Affiliations:** Department of Biotechnology and Life Science, Laboratory of Environmental and Applied Botany, University of Insubria, Via Dunant 3, Varese, 21100, Italy; Department of Biotechnology and Life Science, Laboratory of Environmental and Applied Botany, University of Insubria, Via Dunant 3, Varese, 21100, Italy; Department of Biosciences and Territory, University of Molise, Contrada Fonte Lappone, Pesche (IS), 86090, Italy; Department of Biotechnology and Life Science, Laboratory of Environmental and Applied Botany, University of Insubria, Via Dunant 3, Varese, 21100, Italy; Department of Biosciences and Territory, University of Molise, Contrada Fonte Lappone, Pesche (IS), 86090, Italy; Department of Biotechnology and Life Science, Laboratory of Environmental and Applied Botany, University of Insubria, Via Dunant 3, Varese, 21100, Italy

**Keywords:** abiotic stress interaction, climate extremes, plant resilience, post-stress recovery, thermal stress, water limitation

## Abstract

Climate change is increasing the frequency and intensity of droughts and heatwaves, posing a major threat to forest regeneration and tree survival. Young trees are particularly vulnerable to these stressors due to their limited root systems and reduced physiological buffering capacity. In this study, we investigated the individual and combined effects of progressive drought intensity and elevated temperature on the physiological, morphological and oxidative stress responses of *Populus nigra* seedlings, as well as their capacity to recover following rewatering. Two-year-old seedlings were grown under controlled optimal (20–25 °C) or high (30–35 °C) temperature regimes and subjected to moderate, severe and extreme drought, followed by recovery phases. Plant performance was assessed through measurements of water relations, gas exchange, chlorophyll fluorescence, chlorophyll content, oxidative stress (O₂^−^ accumulation) and aboveground growth traits (i.e., leaf area and biomass, and stem height and biomass). Both drought and heat stress individually impaired plant water status, photosynthetic efficiency and growth, whereas their combination produced synergistic effects, leading to more severe physiological dysfunction, greater oxidative stress and greater growth suppression. Recovery capacity depended strongly on drought severity and temperature conditions. Moderate drought allowed near-complete recovery of physiological function, whereas severe drought resulted in partial recovery, and extreme drought often caused irreversible damage and mortality, particularly under high temperatures. Physiological traits generally recovered faster than morphological traits, indicating that structural growth requires sustained carbon availability beyond initial functional restoration. Multivariate analyses revealed a shift from acquisitive to conservative resource-use strategies under increasing stress, with incomplete reorganization of trait relationships during recovery under high temperature. Our results highlight the vulnerability of young *P. nigra* seedlings to compound drought-heat stress and suggest that future climate extremes may severely constrain seedling establishment, forest regeneration and ecosystem resilience in riparian and temperate forest systems.

## Graphical Abstract

**Graphical Abstract:**
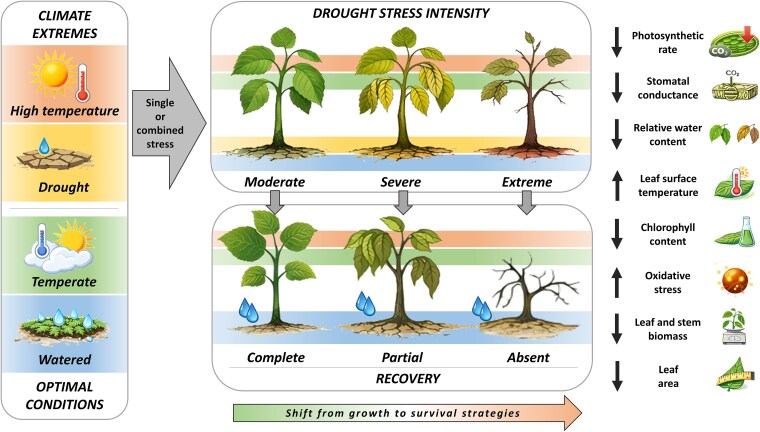

## Introduction

Climate change is driving a rapid intensification of extreme climatic events, particularly droughts and heatwaves, which increasingly co-occur and pose major risks to forest ecosystems. Beyond drought and heatwaves, other climate-driven disturbances, including flooding, windstorms, extreme precipitation and wildfires, also pose substantial risks to plant performance and long-term adaptation, underscoring the increasing pressure exerted by extreme events on terrestrial ecosystems (Chiatante et al. 2015, Ummenhofer and Meehl 2017, Montagnoli et al. 2023). In Europe, future summers are projected to become simultaneously hotter and drier, with more frequent compound drought–heat events that exceed historical extremes (Meehl and Tebaldi 2004, Seneviratne et al. 2006, Sutanto et al. 2025). Observed events in 2003, 2010, 2018, 2022 and 2025 have already demonstrated the ecological consequences of such extremes, including reductions in primary productivity, large-scale stem dehydration, widespread canopy dieback and increased mortality rates across multiple forest types (Ciais et al. 2005, Salomón et al. 2022, Feser et al. 2024, Gugliemi 2025, Knutzen et al. 2025). Young trees are particularly vulnerable because their limited root development, high growth rates and reduced stress-buffering capacity limit their ability to withstand prolonged or intense climatic stress (Wright et al. 2018). Consequently, early seedling establishment has emerged as a critical bottleneck for forest regeneration under future climate conditions (Marchin and Yuan 2023).

Drought is widely recognized as one of the strongest abiotic constraints on tree physiology and growth. Progressive soil drying disrupts plant water status by reducing leaf water potential (LWP), relative water content (RWC) and hydraulic conductivity, which promotes early stomatal closure and limits CO₂ assimilation (Flexas et al. 2004, Tardieu et al. 2018). As stress intensifies, metabolic constraints arise due to impaired electron transport, reduced Rubisco activity, photoinhibition and accumulation of reactive oxygen species, which together reduce photosynthetic efficiency and carbon gain (Chaves et al. 2009). Drought also induces characteristic morphological adjustments that reduce transpirational demand, including declines in leaf expansion, total leaf area (LA), leaf biomass (LB) and stem diameter (SD) growth (Liu and Dickmann 1992, Harvey and van den Driessche 1997, Li et al. 2017). Such structural responses represent critical drought-avoidance strategies but simultaneously reduce the plant’s photosynthetic surface and long-term productivity. Under severe or prolonged water deficit, hydraulic vulnerability becomes a major threat. Cavitation and xylem embolism can occur when water potentials drop below species-specific thresholds, impairing water transport and risking hydraulic failure (Fichot et al. 2015). At the same time, sustained reductions in carbon assimilation contribute to carbon depletion, reduced non-structural carbohydrate pools and weakened stress recovery capacity, processes that can ultimately lead to mortality via interacting hydraulic and carbon-based mechanisms (Menezes-Silva et al. 2019). The interplay of physiological and morphological constraints underpins the complexity of drought responses in young trees and underscores the need for comprehensive, multi-trait assessments across progressive drought intensities.

Heat stress alone profoundly influences plant physiology even when water is not limiting. Temperature increases can stimulate photosynthesis up to an optimal threshold, beyond which further warming impairs physiological processes and reduces photosynthetic performance by destabilizing cellular membranes, decreasing Rubisco activase efficiency and disrupting thylakoid structure (Crafts-Brandner and Salvucci 2000, Wahid et al. 2007). High temperatures also increase leaf-to-air vapor pressure deficit (VPD), thereby enhancing atmospheric evaporative demand and transpiration rates; however, this does not necessarily imply greater stomatal opening, as stomata may partially close under high VPD while residual conductance can still increase (Urban et al. 2017). Heat-induced oxidative stress further exacerbates metabolic disruption by enhancing the production of reactive oxygen species (Mittler 2006). Morphologically, heat can influence biomass allocation patterns, favoring shoot elongation while reducing leaf formation or structural biomass accumulation (Silim et al. 2010). Such shifts reflect plasticity in growth strategies but may reduce structural robustness and resilience to subsequent stress (Samat et al. 2025). Understanding these heat-specific effects is essential for interpreting how elevated temperatures modify drought responses.

When drought and heat occur simultaneously, their effects often interact synergistically rather than additively. Water deficit limits transpiration-driven cooling, causing leaf temperatures to rise more rapidly and severely under high ambient temperatures (Rennenberg et al. 2006). This exacerbates thermal stress, accelerates photoinhibition and intensifies oxidative damage (Suzuki et al. 2014). Combined stress typically causes sharper declines in stomatal conductance, photosynthetic rate, chlorophyll content and photosystem II (PSII) efficiency compared with either stressor alone, while promoting stronger increases in non-photochemical quenching (NPQ) and ROS accumulation. These compounded physiological constraints are mirrored morphologically: combined drought-heat events accelerate leaf senescence, reduce LA and biomass and suppress height and diameter growth more strongly than individual stresses (Hu et al. 2023). Importantly, recovery from combined stress is often incomplete, especially when stress intensity exceeds species-specific safety margins (Kamatchi et al. 2024). For young trees with limited hydraulic safety and carbon reserves, such interactions are likely to determine survival under future climate scenarios.

Poplars (*Populus* spp.) are widely used in stress-physiology research due to their sequenced genome (Tuskan et al. 2006), fast growth, clonal propagation, ecological significance and economic relevance (Jansson and Douglas 2007). *Populus nigra* L., a dominant pioneer of European riparian systems, is particularly exposed to fluctuating water availability, drought episodes and increasingly frequent heatwaves. Young *P. nigra* individuals exhibit reduced photosynthesis, early leaf senescence and heightened vulnerability to cavitation under drought (Regier et al. 2009, Barigah et al. 2013), while heat stress further disrupts photosynthetic function and intensifies oxidative damage (Wahid et al. 2007, Suzuki et al. 2014). Despite extensive research, integrative assessments combining ecophysiology, morphology and oxidative stress markers across increasing drought severities, elevated temperature regimes and subsequent recovery phases remain limited, particularly for early developmental stages, which are critical for regeneration and population persistence.

This study addresses these gaps by evaluating how young *P. nigra* seedlings respond to drought, heat and their combination across progressive stress intensities, and how they recover following rewatering. In particular, we aimed to: (i) assess the individual effects of drought and heat alone on physiological and morphological traits; (ii) determine whether combined drought–heat stress induces additive or synergistic impairments; (iii) evaluate the capacity for post-stress recovery across different drought intensities and temperature regimes; and (iv) identify key traits associated with resilience or vulnerability under combined stress scenarios. More specifically, we hypothesized that (i) physiological and morphological traits of *P. nigra* seedlings would be negatively affected by drought or heat stress when applied individually, with the magnitude of the response increasing with stress intensity and duration. In particular, we expected progressive reductions in LA, LB, stem growth, RWC, LWP, photosynthetic rate, stomatal conductance, chlorophyll content and photochemical efficiency, accompanied by increased leaf surface temperature (LST), NPQ and leaf oxidative stress. In addition, we hypothesized that (ii) the combination of drought and high temperature (HT) would exacerbate the detrimental effects observed under each stress applied alone, resulting in synergistic rather than additive responses. Specifically, we expected stronger impairment of gas exchange, photochemical performance and growth, higher leaf temperatures, enhanced photoprotective energy dissipation, increased superoxide accumulation and an earlier onset of irreversible damage under combined stress compared with single-stress treatments. Furthermore, we hypothesized that (iii) post-stress recovery following rewatering would depend on the severity of the preceding drought stress and on the thermal context, with progressively reduced recovery capacity after moderate, severe and extreme drought. In particular, we hypothesized a rapid and near-complete recovery of plant water status and photosynthetic activity after moderate drought (MD), partial recovery after severe drought (SD) and incomplete or failed recovery after extreme drought, particularly under high-temperature (HT) conditions, with morphological traits showing slower and less complete restoration than physiological traits. Finally, we hypothesized that (iv) multivariate trait coordination would shift from growth- and photosynthesis-dominated strategies under mild stress to survival- and protection-oriented strategies under severe and extreme stress, with recovery phases showing stress-history-dependent trait reorganization. In particular, we expected increasing convergence of traits associated with thermal stress, photoprotection and oxidative damage during drought and incomplete re-expansion of functional trait space during recovery under HT. To test our hypothesis, we grew 2-year-old *P. nigra* seedlings in growth rooms subjected to two temperature regimes (optimal 20–25 °C and high 30–35 °C), combined with three progressive drought levels (moderate, severe, extreme), followed by rewatering periods for recovery. Both morphological and physiological responses, as well as oxidative stress, were measured and monitored.

## Materials and methods

### Plant material and growth room characteristics

The experiment was conducted from April to July 2024 in the Laboratory of Applied and Environmental Botany at the University of Insubria, Varese, Italy. At the end of October 2023, 2-year-old *P. nigra* L. dormant seedlings, originating from seeds, were purchased from a commercial nursery (Società Agricola Forbici SS, Desenzano, Italy, 45°28′13.8" N 10°30′31.0" E). The seedling trays had single wells sized 60 × 60 × 150 mm. At the nursery site, the mean annual temperature is 13.4 °C, with January and July being the coldest and warmest months, respectively. The total annual precipitation is 1045 mm. Upon arrival, the seedlings had a mean stem diameter of 0.36 ± 0.03 cm and a height of 53.7 ± 4.3 cm (mean ± SD).

In February 2024, the seedlings were transplanted in 6.2 L pots (175 × 175 × 250 mm) filled with a growing media composed of a mixture of agricultural loamy-sandy soil (7.9 pH, 2.4% organic carbon content), perlite and peat in a ratio 3:1:1. In detail, the root system of the seedlings was gently washed under tap water to remove the soil, inspected for mechanical, rot or insect damage, placed in the center of the pot and filled with growing media.

The potted seedlings were placed in a growth room with a photoperiod of 16-h light and 8-h dark, a light intensity of 400 μmol m^−2^ s^−1^ at plant height (Valoya RX325HV Solray 385), a temperature of 25/20 °C during the day/night, a relative humidity of 50–65% and an ambient CO_2_ level of 400 μmol mol^−1^. The air temperature and humidity in the growth rooms were monitored throughout the experiment using data loggers (Elitech RC-5, ThermElc TE-02) at 30-min intervals. The VPD was not actively controlled during the experiment, so it fluctuated naturally with temperature. Soil temperature (ST) was manually monitored using a soil probe thermometer (CheckTemp1, Hanna Instruments) at a depth of 20 cm near the tap root for 10 randomly selected seedling pots. Seedlings were then left for 60 days to exit dormancy and acclimate to the growth room’s environmental conditions (until they had fully expanded new leaves). Also, seedlings were irrigated every second day to field capacity, and no fertilization was applied during the experiment.

### Experimental design

**Figure 1:**
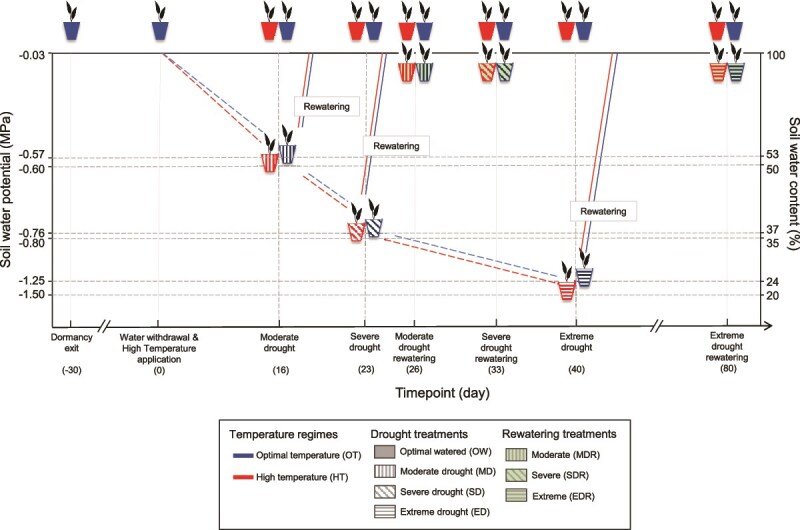
Schematic diagram of the experimental design.

The experimental layout was a randomized block design with two factors: temperature and watering regime. At the beginning of the experiment (April 2024; day 0), seedlings were divided into two different growth rooms and grown at two distinct temperature ranges, defined as optimal (OT, 20–25 °C night/day) and high (HT, 30–35 °C night/day) (Table 1). For each temperature range, water was withheld entirely for a cohort of seedlings that progressively underwent three water-shortage levels: moderate (MD—16 days), severe (SD—23 days) and extreme drought (ED—40 days; Table 1; Figure 1). The rationale for using different levels of water shortage, one after another, was to resemble the natural progressive soil drying. After each water-shortage level was reached, a cohort of water-withdrawal-treated seedlings was subjected to a rewatering (R) treatment to re-establish and maintain optimal soil water content, allowing a recovery period before sampling (10 days for MDR and SDR, and 40 days for EDR). Soil water potential (Ψ_soil_) was regularly measured using gypsum blocks (Delmhorst KS-D1 Digital Soil Moisture Tester) inserted at a depth of 8 cm in the middle of the pot. Six gypsum blocks were used for each treatment. At each sampling point, 9 seedlings were randomly selected from each treatment combination, yielding a total of 216 seedlings across six timepoints. Control seedlings in both temperature ranges were maintained under an optimal watering (OW) regime throughout the experiment. To do so, the control pots were weighed regularly to determine water loss, which was then replenished to maintain a constant soil water content (Table 1; Figure 1).

**Table 1:** Experimental design timing, growth chamber settings, drought levels and corresponding soil water content and potential.

Experimental design		Watering regime	Soil water		Air temperature (°C)	
Day (dd)	Stage description		Content (%)	Potential (MPa)	Optimal (20–25)	High (30–35)
−60 to 0	Acclimatization and exit dormancy	Well-watered	100	−0.03		
0–16	Water withdraws (first level)	Moderate drought (MD)	53	−0.60		
			50	−0.57		
16–23	Water withdraws (second level)	Severe drought (SD)	37	−0.80		
			35	−0.76		
16–26	Rewatering (first level)	Well-watered (MDR)	100	−0.03		
23–33	Rewatering (second level)	Well-watered (SDR)	100	−0.03		
23–40	Water withdraws (third level)	Extreme drought (ED)	24	−1.50		
			20	−1.25		
40–80	Rewatering (third level)	Well-watered (EDR)	100	−0.03		

### Physiological analyses

#### Leaf relative water content, water potential and surface temperature

For the calculation of RWC, four mature leaves from each seedling were sampled and immediately weighed to determine fresh weight (FW). Then, the leaves were placed in distilled water in the dark for 24 h to allow water uptake and swelling, and to determine their turgid weight (TW). The samples were then oven-dried at 70 °C until constant weight, and their dry weight (DW) was recorded.

Pre-dawn LWP was determined using a Scholander pressure chamber (Skye SKPM 1400/40, Skye Instruments Ltd, UK) on two fully expanded leaves from the upper part of the crown (usually the third and fourth mature leaves under the apex) of nine seedlings. The measurements were performed 5–6 h before the lights were turned on.

To assess LST, thermal images were obtained using a FLIR T420 infrared camera (Flir Systems, Wilsonville, OR, USA). The system incorporates a focal-plane-array uncooled microbolometer (detector type) with a spectral range of 7.5–13 μm, 320 × 240 pixels and a built-in 25° lens. Emissivity was set at 0.95 to view leaves. At each sampling point, thermal images were captured for at least 10 seedlings per treatment combination. The overall temperature of the top fully expanded leaf per plant was determined at a 2 m distance from the plant. Images were subsequently analyzed to determine temperature using the FLIR software. The software allows a polygonal area to be drawn along the edges of the leaf surface to measure the overall leaf temperature.

#### Photosynthetic rate, leaf gas exchange and water-use efficiency

Using a portable photosynthesis system (Ciras-2, PP Systems Inc., Amesbury, USA), net photosynthetic rate (Pn), stomatal conductance (Gs) and transpiration rate (Tr) were measured on the second fully expanded leaf under the apical bud for nine seedlings in each treatment combination. Measurements were taken ~5 min after the leaf was acclimated in the cuvette, with three readings taken within 1 min. The cuvette parameters during the measurement were set as follows: photosynthetically active radiation, 400 μmol m^−2^ s^−1^; flow rate, 220 μmol s^−1^; CO_2_ concentration, 400 μmol mol^−1^; and temperature, 25 or 35 °C, depending on the treatment. Finally, photosynthetic water-use efficiency (WUE) was defined as the ratio of the rate of Pn to Tr.

#### Chlorophyll content and leaf fluorescence

Non-destructive estimation of leaf chlorophyll content (LCC) was determined at each sampling point, using an optical pigment sensor (Dualex Scientific, FORCE-A, Orsay, France). Measurements were performed on the second fully expanded leaf under the apical bud in nine seedlings. Chlorophyll fluorescence was measured during dark and light periods using a portable pulse-modulated fluorometer (OS1-FL, Opti-Sciences, Inc., USA). Minimal fluorescence (F0) was measured on dark-adapted leaves during the dark period. After F0 determination, maximal fluorescence yield (Fm) of the dark-adapted leaves was recorded after exposing them to a saturating pulse of white light (800 ms at ~15,000 μmol photons m^−2^ s^−1^). Values of F0 and Fm were used to calculate maximal photochemical efficiency of PSII (Fv/Fm), where Fv is variable fluorescence (Fv = Fm – F0). Quantum yield of PSII (ФPSII) was measured during the light period. Fluorescence at steady state (Fs) and maximal fluorescence yield (Fms) of the light-adapted leaves were recorded after exposing leaves to a saturated pulse of white light (800 ms at ~15,000 μmol photons m^−2^ s^−1^). Values of Fs and Fms were used to calculate ФPSII and NPQ, as described in Murchie and Lawson (2013).

### In situ localization and quantitative analysis of superoxide anion radicals

Stress-induced generation of superoxide anion radicals (O_2_^−^) was detected by monitoring the reduction of nitro-blue tetrazolium (NBT, ChemCruz, Santa Cruz Biotechnology, Inc.). The NBT was freshly prepared in 20 mM potassium phosphate buffer (pH 6.1) and diluted to 0.1 mg mL_−1_. For in situ staining of O_2_^−^, 2 cm–diameter leaf disks were sampled from the first fully expanded leaf and immediately infiltrated under vacuum (−5 PSI/−0.03 Pa) with NBT solution for 10 min. Incubation was carried out on a laboratory shaker for 2 h in the dark at room temperature. For the negative control, disks were incubated with 20 mM potassium phosphate buffer. The reaction was stopped by transferring the disks to distilled water. The chlorophyll was bleached by boiling twice in 96% ethanol for 15 min, followed by 30% ethanol for 30 min or until complete removal. Three randomly selected observation fields (2.24 mm^2^) per disk were captured under a light microscope (Olympus BX63, Olympus Life Sciences) at 4× magnification. Areas near the leaf edge were avoided as higher O_2_^−^ concentrations were clearly detectable due to the cut made to obtain leaf disks. Quantitative analysis was conducted by scanning the pixels of blue-stained spots and the entire disk area with WinRhizo. Generation of O_2_^−^ was expressed as the percentage of pixels in the stained area versus the total number of pixels from the disk.

### Morphological analysis

At each sampling point, nine seedlings were randomly sampled for each treatment combination and dissected to measure aboveground morphological traits. All leaves present on the plant at the time of harvest were collected, including both leaves developed pre-treatment and new leaves formed during the treatment. Total LA was measured by scanning the leaves (Epson Expression 12000 XL) and analyzing the obtained images with WinRhizo Pro V. 2007d (Regent Instruments Inc., Quebec). Afterward, the leaves were oven-dried at 70 °C until constant weight to obtain the LB. The SD was measured at the base of the seedling using a high-precision digital caliper (MT-112503) with a 0.1 mm resolution. Stem height (SH) was measured as the distance between the stem base and the shoot apex with a meter stick. Finally, the seedling’s stem was oven-dried at 70 °C until constant weight to obtain the stem biomass (SB) and calculate the aboveground biomass (AB).

### Statistical analyses

All statistical analyses were conducted in R v4.2.2. To assess differences among treatments for physiological and morphological parameters (RWC, LWP, leaf temperature, photosynthetic rate, transpiration rate, stomatal conductance, WUE, chlorophyll content, maximum quantum efficiency of PSII, ФPSII, NPQ, O_2_^−^ quantification, LA, LB, SH, SD, SB, ST), the Kruskal–Wallis non-parametric test was applied. When significant differences were found, Dunn’s post hoc test was used for pairwise comparisons. To evaluate the main and interactive effects of the watering and temperature regimes, an Aligned Rank Transformation ANOVA (ART-ANOVA) was conducted separately for the drought phase (MD, SD, ED) and the recovery phase (MDR, SDR, EDR). In this model, both factors were treated as fixed effects. To evaluate the main and interactive effects of temperature regime and time, an ART-ANOVA was conducted for well-watered plants grown at OT and HT, with temperature regime and time as fixed factors. Statistical significance was determined at *P* < 0.05. In addition, a principal component analysis (PCA) was performed on trait data from six sampling dates, separately, to characterize multivariate physiological and morphological responses across drought intensities and subsequent recovery in both OT and HT conditions.

### Use of AI-assisted technologies

During the preparation of this work, the authors used ChatGPT and Grammarly to improve the manuscript’s readability and language. After using this tool, the authors reviewed and edited the content of the published article.

## Results

In our experiment, the ED treatment led to complete leaf senescence, which prevented all leaf-based measurements (e.g., RWC, leaf temperature, LWP, net photosynthetic rate, transpiration rate, stomatal conductance, WUE, maximal photochemical efficiency of PSII, ФPSII, NPQ, chlorophyll content, quantification of O_2_^−^, LA, LB) in the drought-treated plants, whereas well-watered plants remained fully measurable at the same sampling point. Moreover, only half of the plants under HT conditions recovered from ED, exhibiting a new flush of growth. The other half showed no signs of recovery, with stems completely dehydrated and no new growth observed during the 40-day rewatering period. In contrast, all plants in the OT regime recovered from ED.

### Leaf relative water content, water potential and surface temperature

The results of the ART-ANOVA analysis revealed that, during the drought phase, both drought (*D*) and temperature (*T*) had highly significant effects (*P* < 0.001) on RWC, LWP and LST (Supplementary Table S1 available as Supplementary Data at *Tree Physiology* Online). The interaction between drought and temperature (*D* × *T*) was very significant for RWC (*P* < 0.01) and highly significant for LST (*P* < 0.001), while showing no significant effect on LWP. The data from the rewatering phase revealed that the severity of the prior drought, the temperature regime and the interaction *D* × *T* had significant and highly significant effects on RWC, LWP and LST.

**Figure 2:**
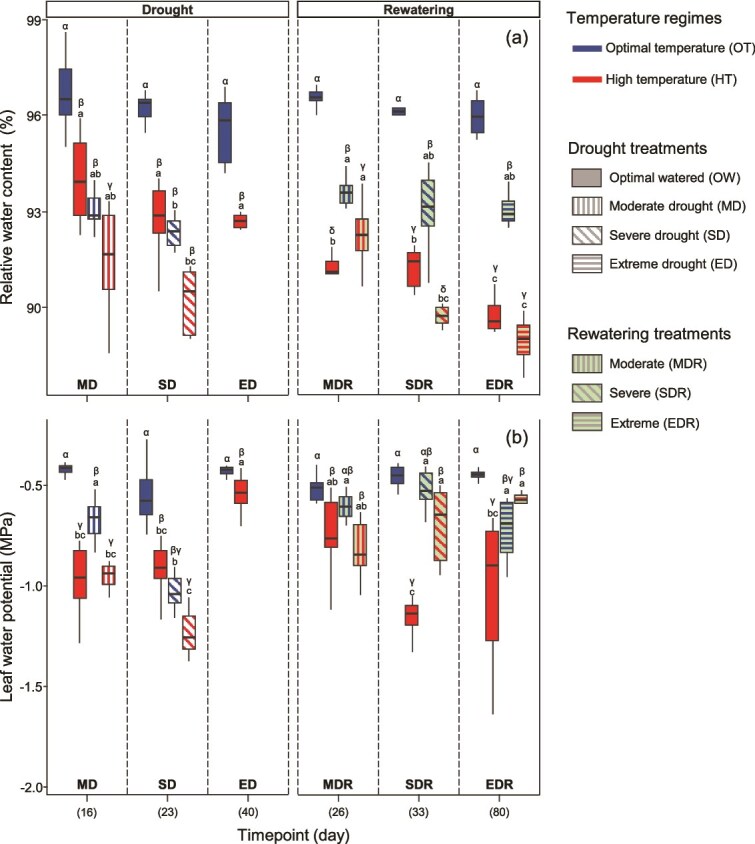
Dynamics of (a) relative water content (RWC; %) and (b) leaf water potential (LWP; MPa) in *P. nigra* seedlings in response to drought, heat and their combination across six experimental sampling points. Timepoints correspond to progressive drought stages (moderate (MD), severe (SD), extreme (ED)) and the following rewatering stages (moderate drought rewatering (MDR), severe drought rewatering (SDR), extreme drought rewatering (EDR)). Within each stage, different groups of plants were subjected to optimal (OT) or high temperature (HT) and to optimal watering (OW) or drought (MD, SD, ED). Boxes represent ~50% of the observations (*n* = 9 biological replicates), while lines extending from each box represent the upper and lower 25% of the distribution. Within each box, the solid horizontal line represents the median value. Latin letters indicate significant differences (*P* < 0.05) within each treatment across drought and rewatering stages. Greek letters indicate significant differences among treatments within the same stage.

**Figure 3:**
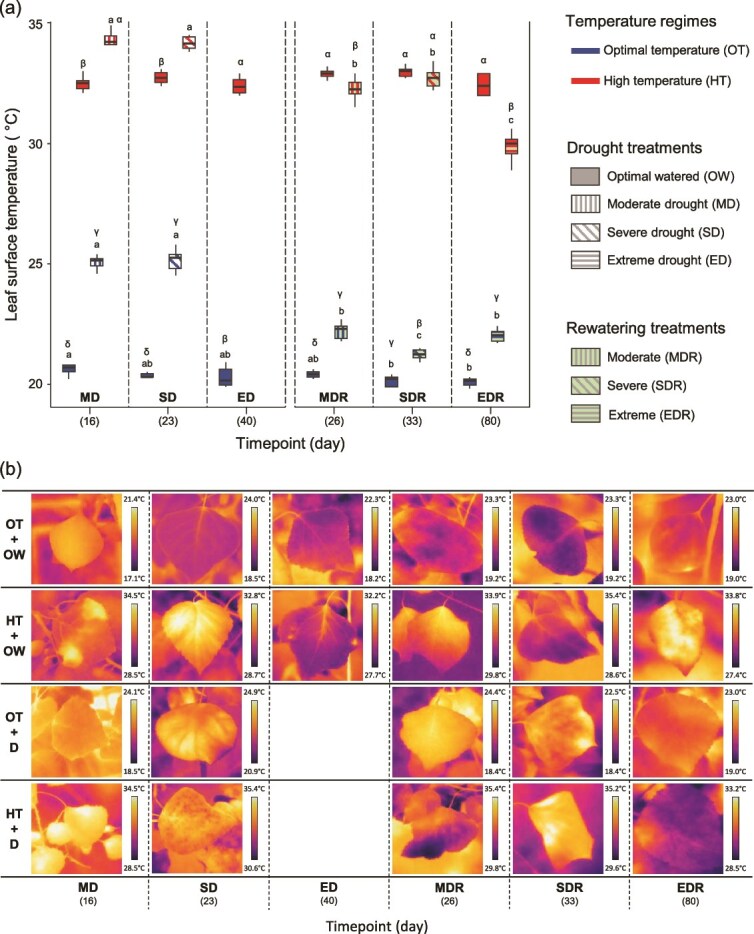
(a) Dynamics of leaf surface temperature (LST; °C) and (b) representative thermal images of leaves of *P. nigra* seedlings in response to drought, heat and their combination across six experimental sampling points. Timepoints correspond to progressive drought stages (moderate (MD), severe (SD), extreme (ED)) and the following rewatering stages (moderate drought rewatering (MDR), severe drought rewatering (SDR), extreme drought rewatering (EDR)). Within each stage, different groups of plants were subjected to optimal (OT) or high temperature (HT) and to optimal watering (OW) or drought (D). Boxes represent ~50% of the observations (*n* = 9 biological replicates), while lines extending from each box represent the upper and lower 25% of the distribution. Within each box, the solid horizontal line represents the median value. Latin letters indicate significant differences (*P* < 0.05) within each treatment across drought and rewatering stages. Greek letters indicate significant differences among treatments within the same stage.

Under OW conditions, HT-treated plants showed significantly lower RWC and LWP values (Figure 2a and b) and higher LST values (Figure 3a and b) than plants subjected to OT conditions at all measured timepoints.

At OT, plants subjected to MD showed a significant decline in RWC and LWP when compared with OW plants, and a further drop was observed in plants subjected to SD (Figure 2a and b), while LST increased significantly above OW plants in both MD and SD plants (Figure 3a). At HT, MD plants showed a significant decrease in RWC, reaching their lowest recorded values (Figure 2a), LST significantly increased, reaching the highest recorded values (Figure 3a), while LWP showed no statistically significant differences. On the contrary, SD plants showed no significant reduction in RWC (Figure 2a) but exhibited a reduction in LWP (Figure 2b) and an increase in LST (Figure 3a).

In MD- and SD-rewatered plants (MDR and SDR), the RWC values in OT plants were slightly higher but not significant (*P* > 0.05) than those observed in plants in the respective drought stage (MD or SD), though significantly lower than those of OW plants within the rewatering stages MDR or SDR (Figure 2a). While not comparable to ED plants, EDR plants showed lower RWC values than the respective OW plants. A similar trend was observed in plants subjected to HT, with no significant differences between the rewatered plants MDR and SDR and the respective drought plants MD and SD. On the contrary, during the rewatering stage, HT MDR plants showed significantly higher RWC values than the respective OW plants, whereas SDR and EDR plants showed lower values when compared with the respective OW plants (Figure 2a).

Rewatering led to diverse LWP outcomes across the different drought levels and temperature treatments (Figure 2b). At the OT, MDR and SDR plants recovered the LWP completely and reached values similar to those of the respective OW plants, while EDR plants showed lower values. In contrast, when HT plants were considered, LWP showed similar values between rewatered and OW plants at the MDR stage, whereas SDR and EDR plants showed higher values than their respective OW plants (Figure 2b).

Optimal-temperature (OT) MDR, SDR and EDR plants showed significantly higher LST values than the respective OW plants (Figure 3a), whereas MDR and SDR showed lower values than MD and SD plants, respectively. On the contrary, HT MDR and EDR plants exhibited lower LST values than the respective OW plants, whereas SDR plants showed a statistically insignificant decrease. Furthermore, both MDR and SDR plants showed significantly lower values than MD and SD plants (Figure 3a).

### Photosynthetic rate, leaf gas exchange and water-use efficiency

The results of the ART-ANOVA analysis revealed that, during the drought phase, drought had a very significant effect (*P* < 0.01) on net photosynthetic rate (Pn), transpiration rate (Tr) and stomatal conductance (Gs) but not photosynthetic WUE (Supplementary Table S1 available as Supplementary Data at *Tree Physiology* Online). Temperature (*T*) had a highly significant effect (*P* < 0.001) on Pn, Tr and WUE and a significant effect on Gs (*P* < 0.05). The interaction between drought and temperature (*D* × *T*) was significant for Pn (*P* < 0.05) and highly significant for Tr and Gs (*P* < 0.001), while it had no significant effect on WUE. The data from the rewatering phase revealed that the severity of the prior drought significantly affected Gs (*P* < 0.05) but not Pn, Tr or WUE. The temperature regime had a highly significant effect on Pn, Tr and WUE (*P* < 0.001) and a very significant effect on Gs (*P* < 0.01). A significant and highly significant interaction *D* × *T* was observed for Pn (*P* < 0.001), Tr (*P* < 0.001) and Gs (*P* < 0.05), while no significant interaction was found for WUE.

**Figure 4:**
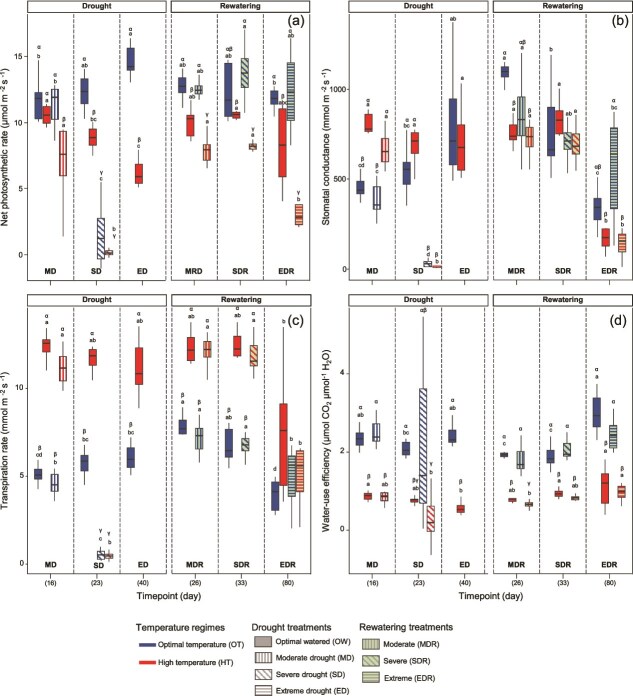
Dynamics of (a) net photosynthetic rate (Pn; μmol m^−2^ s^−1^), (b) stomatal conductance (Gs; mmol m^−2^ s^−1^), (c) transpiration rate (Tr; mmol m^−2^ s^−1^) and (d) water-use efficiency (WUE; μmol CO_2_ μmol^−1^ H_2_O) in *P. nigra* seedlings leaves in response to drought, heat and their combination across six experimental sampling points. Timepoints correspond to progressive drought stages (moderate (MD), severe (SD), extreme (ED)) and the following rewatering stages (moderate drought rewatering (MDR), severe drought rewatering (SDR), extreme drought rewatering (EDR)). Within each stage, different groups of plants were subjected to optimal (OT) or high temperature (HT) and to optimal watering (OW) or drought (MD, SD, ED). Boxes represent ~50% of the observations (*n* = 9 biological replicates), while lines extending from each box represent the upper and lower 25% of the distribution. Within each box, the solid horizontal line represents the median value. Latin letters indicate significant differences (*P* < 0.05) within each treatment across drought and rewatering stages. Greek letters indicate significant differences among treatments within the same stage.

Under OW conditions, HT-treated plants showed significantly lower Pn values than plants subjected to OT conditions at four of the six timepoints measured (days 23, 26, 40 and 80; Figure 4a). On the contrary, Gs showed contrasting values: significantly higher at day 16 and lower at day 26, with no significant differences at the subsequent timepoints (Figure 4b). The Tr showed significantly higher values in HT plants across five timepoints (Figure 4c), whereas WUE exhibited lower values at all timepoints (Figure 4d).

At OT, plants subjected to MD showed no statistically significant differences in Pn, Gs, Tr or WUE compared with OW plants (Figure 4a-d). In contrast, plants subjected to SD showed significant decreases in all parameters, with values approaching zero, except for WUE. At HT, both MD and SD plants showed a significant Pn decrease compared with OW plants, while a Gs and Tr decrease was observed only in SD plants. No significant differences in WUE were observed.

In MD-rewatered (MDR) plants, those subjected to OT (Figure 4a-d, blue boxes) showed significantly higher Gs and Tr values than MD plants, while showing similar Pn and WUE values and no statistically significant differences compared with the respective OW plants. In contrast, MDR plants subjected to HT conditions (Figure 4a-d, red boxes) showed no differences relative to the respective MD plant but exhibited lower Pn and WUE than the respective OW plants.

Among the severe drought–rewatered (SDR) plants, plants subjected to OT (Figure 4a-d, blue boxes) showed significantly higher Pn, Gs and Tr values than SD plants, while showing no statistically significant differences compared with the respective OW plants. Similarly, SDR plants subjected to high-temperature conditions (Figure 4a-d, red boxes) showed significantly higher Pn, Gs and Tr values than SD plants but exhibited lower Pn values than the respective OW plants.

When ED-rewatered (EDR) plants were compared with the respective OW plants, no significant differences in any measured traits were observed at either temperature condition, except for HT plants, which exhibited lower Pn values (Figure 4a).

### Chlorophyll content and leaf fluorescence

The results of the ART-ANOVA analysis revealed that, during the drought phase, both drought (*D*) and temperature (*T*) had a highly significant effect (*P* < 0.001) on LCC, maximal photochemical efficiency of PSII (Fv/Fm), ФPSII and NPQ (Supplementary Table S1 available as Supplementary Data at *Tree Physiology* Online), with the only exception of *D* having a very significant effect (*P* < 0.01) on Fv/Fm. The interaction between drought and temperature (*D* × *T*) was significant for Fv/Fm (*P* < 0.05), very significant for ФPSII (*P* < 0.01) and highly significant for LCC and NPQ (*P* < 0.001). The data from the rewatering phase revealed that the severity of the prior drought had a highly significant effect (*P* < 0.001) on LCC, Fv/Fm and NPQ, but not ФPSII. The temperature regime had a highly significant effect (*P* < 0.001) on all traits (LCC, Fv/Fm, ФPSII and NPQ). Highly significant interactions *D* × *T* were observed for LCC and NPQ (*P* < 0.001), while no significant interactions were found for Fv/Fm and ФPSII.

**Figure 5:**
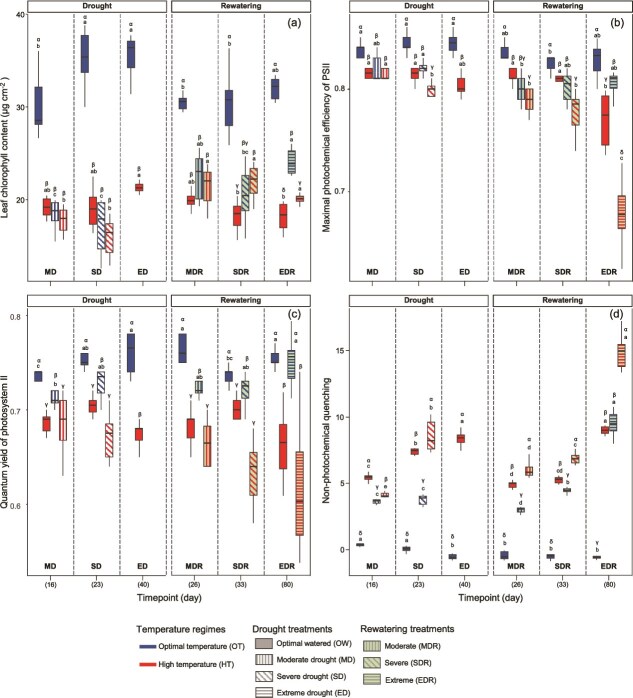
Dynamics of (a) leaf chlorophyll content (LCC; μg cm^−2^), (b) maximal photochemical efficiency of PSII (Fv/Fm), (c) quantum yield of photosystem II (ФPSII) and (d) non-photochemical quenching (NPQ) in *P. nigra* seedlings in response to drought, heat and their combination across six experimental sampling points. Timepoints correspond to progressive drought stages (moderate (MD), severe (SD), extreme (ED)) and the following rewatering stages (moderate drought rewatering (MDR), severe drought rewatering (SDR), extreme drought rewatering (EDR)). Within each stage, different groups of plants were subjected to optimal (OT) or high temperature (HT) and to optimal watering (OW) or drought (MD, SD, ED). Boxes represent ~50% of the observations (*n* = 9 biological replicates), while lines extending from each box represent the upper and lower 25% of the distribution. Within each box, the solid horizontal line represents the median value. Latin letters indicate significant differences (*P* < 0.05) within each treatment across drought and rewatering stages. Greek letters indicate significant differences among treatments within the same stage.

Under OW conditions, HT-treated plants showed significantly lower LCC, Fv/Fm and ФPSII values and higher NPQ values than plants subjected to OT conditions at all measured timepoints (Figure 5a-d).

At optimal temperature, plants subjected to MD and SD showed significant declines in LCC, Fv/Fm, ФPSII and NPQ compared with OW plants (Figure 5a-d). At HT, consistently low LCC and ФPSII values were observed in both MD and SD plants, with no significant differences compared with OW plants (Figure 5a and c). In contrast, MD plants showed lower NPQ values than OW plants, while SD plants showed lower Fv/Fm and higher NPQ than their respective OW plants (Figure 5b and d).

In MDR plants, those subjected to OT (Figure 5a-d, blue boxes) showed significantly lower LCC, Fv/Fm and ФPSII values, while exhibiting higher NPQ values than the respective OW plants. Compared with MD plants, higher LCC and lower NPQ were observed, while Fv/Fm and ФPSII showed no statistically significant differences. Similarly, MDR plants subjected to HT conditions (Figure 5a-d, red boxes) exhibited lower Fv/Fm and higher NPQ values than the respective OW plants, while no differences were observed in LCC and ФPSII. Compared with MD plants, higher LCC and NPQ, lower Fv/Fm and no significant differences in ФPSII were observed.

Among the severe drought rewatered plants (SDR), plants subjected to OT (Figure 5a-d, blue boxes) showed significantly lower LCC, Fv/Fm and ФPSII values, while showing higher NPQ values, compared with the respective OW plants. Compared with SD plants, higher NPQ was observed, while LCC, Fv/Fm and ФPSII showed no statistically significant differences. The SDR plants subjected to Ht conditions (Figure 5a-d, red boxes) showed significantly higher LCC and NPQ, lower Fv/Fm and no significant differences in ФPSII, compared with the respective OW plants. Compared with SD plants, higher LCC and lower NPQ were observed, while Fv/Fm and ФPSII showed no statistically significant differences.

When EDR plants were compared with the respective OW plants, lower LCC and Fv/Fm and higher NPQ were observed in plants subjected to the OT regime (Figure 5a-d, blue boxes). In contrast, higher LCC and NPQ and lower Fv/Fm were observed in plants subjected to HT conditions (Figure 5a-d, red boxes).

### Superoxide anion radicals

The results of the ART-ANOVA analysis revealed that, during the drought phase, both drought (*D*; *P* < 0.05), temperature (*T*; *P* < 0.001) and their interaction (*D* × *T*; *P* < 0.001) significantly affected superoxide anion radical (O_2_^−^) accumulation (Supplementary Table S1 available as Supplementary Data at *Tree Physiology* Online). The data from the rewatering phase revealed that the severity of the prior drought significantly (*P* < 0.05) affected O_2_^−^ accumulation, while no significant effect was observed for temperature. Furthermore, the *D* × *T* interaction was also significant (*P* < 0.05).

**Figure 6:**
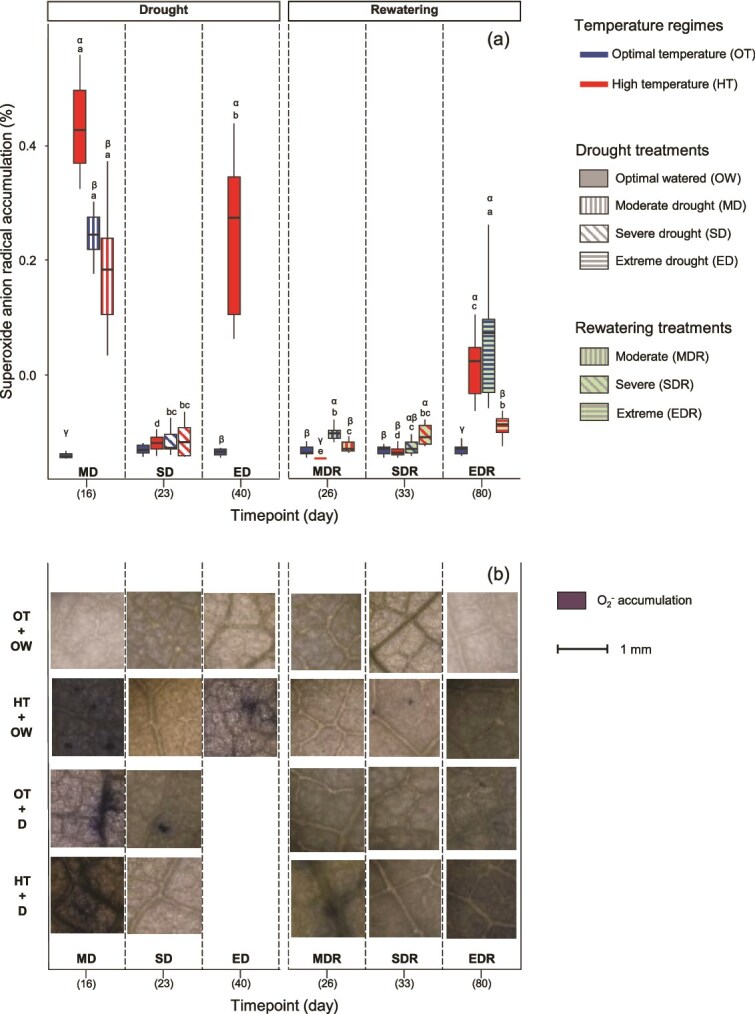
(a) Dynamics of superoxide anion radical (O_2_^−^; %) accumulation and (b) representative images of leaves of *P. nigra* seedlings in response to drought, heat and their combination across six experimental sampling points. Timepoints correspond to progressive drought stages (moderate (MD), severe (SD), extreme (ED)) and the following rewatering stages (moderate drought rewatering (MDR), severe drought rewatering (SDR), extreme drought rewatering (EDR)). Within each stage, different groups of plants were subjected to optimal (OT) or high temperature (HT) and to optimal watering (OW) or drought (MD, SD, ED). Boxes represent ~50% of the observations (*n* = 9 biological replicates), while lines extending from each box represent the upper and lower 25% of the distribution. Within each box, the solid horizontal line represents the median value. Latin letters indicate significant differences (*P* < 0.05) within each treatment across drought and rewatering stages. Greek letters indicate significant differences among treatments within the same stage.

Under OW conditions, HT-treated plants showed significantly higher O_2_^−^ accumulation than plants subjected to OT conditions at three of the six timepoints measured (days 16, 40 and 80) while showing no differences at days 23 and 33 and lower values at day 26 (Figure 6a and b) .

At OT conditions, plants subjected to MD showed a significant increase in O_2_^−^ accumulation, while plants subjected to SD showed no differences compared with OW plants (Figure 6a). At HT, MD plants showed significantly lower O_2_^−^ accumulation than the respective OW plants.

In MDR plants, both plants subjected to OT and HT showed significantly higher O_2_^−^ accumulation values than the respective OW plants. Compared with MD plants, significantly lower values were observed under both temperature regimes (Figure 6a).

Among SDR plants, only plants subjected to HT showed higher O_2_^−^ accumulation values than the respective OW plants, while no differences were observed at OT and compared with SD plants (Figure 6a).

When EDR plants were compared with the respective OW plants, significantly higher O_2_^−^ accumulation values were observed at OT. On the contrary, significantly lower O_2_^−^ accumulation values were observed at HT (Figure 6a).

### Plant morphology

The results of the ART-ANOVA analysis revealed that, during the drought phase, drought had a highly significant effect (*P* < 0.001) on LA, LB, SB and SH (Supplementary Table S1 available as Supplementary Data at *Tree Physiology* Online). Temperature (*T*) had a highly significant effect (*P* < 0.001) only on SH, while no significant effect was observed on LA, LB and SB. The interaction between drought and temperature (*D* × *T*) was significant for LB (*P* < 0.05) and highly significant for SB (*P* < 0.001), while it had no significant effect on LA and SH. The data from the rewatering phase revealed that the severity of the prior drought significantly affected all measured parameters, with highly significant interaction for LA, LB and SB and very significant interactions for SH. The temperature regime had a highly significant effect on SH (*P* < 0.001) and a significant effect on LA and SB (*P* < 0.01) but no significant effect on LB. A highly significant interaction *D* × *T* was observed for LA (*P* < 0.001) and a very significant one for LB (*P* < 0.01) and SB (*P* < 0.01), while no significant interaction was found for SH.

**Figure 7:**
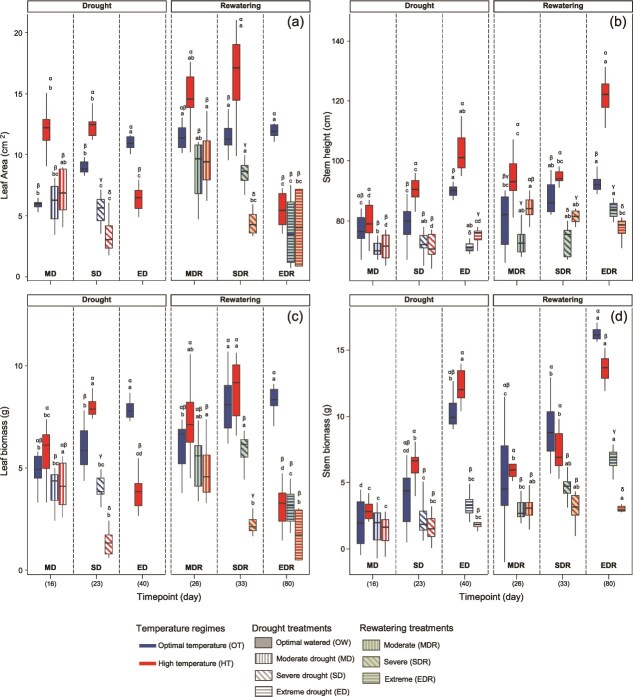
Dynamics of (a) leaf area (LA; cm^2^), (b) stem height (SH; cm), (c) leaf biomass (LB; g) and (d) stem biomass (SB; g) in *P. nigra* seedlings in response to drought, heat and their combination across six experimental sampling points. Timepoints correspond to progressive drought stages (moderate (MD), severe (SD), extreme (ED)) and the following rewatering stages (moderate drought rewatering (MDR), severe drought rewatering (SDR), extreme drought rewatering (EDR)). Within each stage, different groups of plants were subjected to optimal (OT) or high temperature (HT) and to optimal watering (OW) or drought (MD, SD, ED). Boxes represent ~50% of the observations (*n* = 9 biological replicates), while lines extending from each box represent the upper and lower 25% of the distribution. Within each box, the solid horizontal line represents the median value. Latin letters indicate significant differences (*P* < 0.05) within each treatment across drought and rewatering stages. Greek letters indicate significant differences among treatments within the same stage.

Under OW conditions, HT-treated plants showed significantly higher LA than plants subjected to OT conditions at three of the six timepoints measured (days 16, 23 and 33), while lower values were observed on days 40 and 80 (Figure 7a). Leaf biomass (LB) showed higher values only on day 23, no differences on days 16, 26 and 33 and lower values on days 40 and 80 (Figure 7c). On the contrary, SH showed no differences only at day 16; at subsequent timepoints, higher SH values were consistently observed in HT plants compared with OT plants (Figure 7b). Finally, SB showed no differences across all timepoints measured, with the only exception at day 80, where lower SB values were observed in HT plants (Figure 7d).

At OT, plants subjected to MD showed no statistically significant differences in LA, LB, SH and SB compared with OW plants (Figure 7a-d). At HT, MD plants exhibited a decrease in LA and SH while showing no differences in LB and SB. Plants subjected to SD showed a decrease in LA and LB at OT, while at HT, a significant decrease in all parameters was observed. Furthermore, plants subjected to ED showed a decrease in SH at optimal temperature conditions and in SH and SB at HT conditions.

In MDR plants, plants subjected to OT (Figure 7a-d, blue boxes) showed no statistically significant differences compared with both the respective OW plants and MD plants. On the contrary, MDR plants subjected to HT conditions, (Figure 7a-d, red boxes) exhibited lower LA, LB and SB values than the respective OW plants. Compared with MD plants, higher SH and SB values were observed, while no statistically significant differences were observed for LA and LB.

Among the SDR plants, plants subjected to OT (Figure 7a-d, blue boxes) showed significantly lower LA, LB, SH and SB than the respective OW plants. Compared with SD plants, higher LA, LB and SB were observed. The SDR plants subjected to HT conditions (Figure 7a-d, red boxes) showed significantly lower LA, LB, SH and SB than the respective OW plants. Compared with SD plants, higher SH values were observed, while no statistically significant differences were observed for the other parameters.

When EDR plants were compared with the respective OW plants, lower LA, LB, SH and SB were observed in plants subjected to the OT regime (Figure 7a-d, blue boxes). Compared with ED plants, higher SB values were observed. Similarly, lower SH and SB values were observed in plants subjected to HT conditions (Figure 7a-d, red boxes). Compared with ED plants, higher SB values were observed, while no statistically significant differences were observed for SH.

### Trait responses during drought and rewatering phases

**Figure 8:**
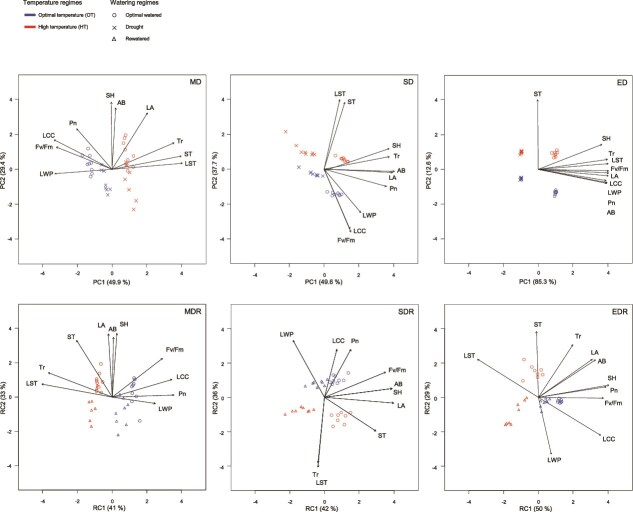
Principal component analysis (PCA) plots of physiological parameters and morphological traits in *P. nigra* seedlings across six experimental timepoints. Each PCA corresponds to one of the following stages: moderate drought (MD), severe drought (SD), extreme drought (ED), moderate drought rewatering (MDR), severe drought rewatering (SDR), extreme drought rewatering (EDR). Abbreviations: LWP (leaf water potential); Fv/Fm (maximal photochemical efficiency of PSII); LCC (leaf chlorophyll content); Pn (net photosynthetic rate); SH (stem height); AB (aboveground biomass); LA (leaf area); Tr (transpiration rate); ST (soil temperature); LST (leaf surface temperature).

The PCA was used to investigate treatment-specific variation in physiological, morphological and biochemical traits under increasing drought intensity levels, namely, moderate (MD), severe (SD) and extreme (ED) (Figure 8). Across all three stages, the first two principal components (PC1 and PC2) explained the majority of the total variance, enabling the effective separation of stress treatments. Across drought stages, PC1 represented the dominant axis of physiological performance and stress intensity, while PC2 captured secondary trait variation whose biological interpretation changed with increasing drought severity. Specifically, under MD, PC1 (49.9%) was positively related to LST, ST and transpiration rate (Tr) while negatively associated with LWP, LCC, maximal photochemical efficiency of PSII (Fv/Fm) and net photosynthetic rate (Pn). PC2 (29.4%) was represented by growth-related traits, including SH, AB and LA. During severe drought, PC1 (49.6%) remained associated with photosynthetic rate, transpiration rate, SH and LA, while rising LST and ST and declining chlorophyll content and PSII efficiency shaped PC2 (37.7%). Under ED, PC1 alone explained a dominant 85.3% of the variation, associated with photosynthetic and transpiration rates, LWP, chlorophyll content, PSII efficiency and growth parameters. PC2 (12.6%) captured ST and, to a lesser extent, SH and transpiration rate.

The PCA during rewatering phases explained 74.3% (rewatering from MD; MDR), 77.6% (rewatering from SD; SDR) and 79.1% (rewatering from ED; EDR) of the total variance across the first two components, hereafter referred to as recovery component 1 (RC1) and recovery component 2 (RC2). During MDR, RC1 (41.1%) was associated with variation in photosynthetic capacity, chlorophyll content, Fv/Fm, and dry mass of leaves and stems, consistent with recovery-related trait variation. In comparison, RC2 (33.2%) captured variation in structural parameters such as LA and SH. In severe drought rewatering, RC1 (41.6%) was driven by biomass and photosynthetic traits, while RC2 (36.1%) captured variation in LWP, transpiration rate and leaf temperature. In EDR, RC1 (50%) was associated with variation in photosynthetic and biomass-related traits, including photosynthetic rate, chlorophyll, Fv/Fm, LA and LB. RC2 (29.1%) was associated with LWP, SH and transpiration. The HT EDR plants clustered at negative values along both recovery components, indicating persistent physiological and morphological impairment despite rewatering. In contrast, OT EDR plants showed intermediate scores along RC1, indicating partial recovery of photosynthetic function, but remained constrained along RC2, reflecting incomplete restoration of water regulation.

## Discussion

This study investigated the physiological and morphological responses of young *P. nigra* seedlings to varying drought intensities (moderate, severe and extreme) and to elevated temperature, both alone and in combination. Additionally, we assessed seedlings’ recovery capacity following rewatering to identify key traits involved in stress resilience and post-stress recovery.

### The individual effects of drought and heat impact physiological and morphological traits

The results clearly support our first hypothesis that both drought and elevated temperatures individually impair physiological and morphological traits in young *P. nigra* seedlings. Under moderate and severe drought at OT, seedlings exhibited progressive reductions in RWC, LWP, photosynthetic performance such as net photosynthetic rate (Pn), maximal photochemical efficiency of PSII (Fv/Fm) and ФPSII, and reduction of growth parameters such as LA and LB, SH and SB, consistent with water limitation constraining cellular hydration, nutrient transport and photosynthesis. From a physiological perspective, the reduction in RWC and the increasingly more negative LWP indicate a loss of tissue hydration and declining plant water status as soil water availability decreased. This hydraulic limitation is known to trigger stomatal closure, reducing CO₂ diffusion into the leaf and thereby constraining carbon assimilation (Flexas et al. 2004, Tardieu et al. 2018). The observed decline in Pn under drought is therefore consistent with a primarily stomatal limitation during moderate stress, which progressively shifts toward non-stomatal limitations under more severe water deficit, including biochemical constraints on the Calvin cycle and photochemical dysfunction (Flexas and Medrano 2002). The parallel decrease in Fv/Fm and ΦPSII suggests that drought not only restricted leaf gas exchange but also impaired the efficiency of the photosynthetic apparatus, likely due to photoinhibition, reduced electron transport capacity and damage to photosystem II reaction centers (Wang et al. 2018). Morphologically, the reduction in LA and biomass reflects both inhibited leaf expansion and enhanced leaf senescence under water deficit, strategies that reduce water loss but also limit light interception and carbon gain (Legg et al. 1979). Similarly, the decline in SH and biomass indicates suppressed primary growth, which is strongly dependent on turgor-driven cell elongation (Shao et al. 2008). Together, these responses demonstrate a coordinated shift from growth-oriented to survival-oriented functioning as water stress intensifies (Chaves et al. 2009). The severity of impairment increased with drought intensity, confirming that young seedlings’ physiological and morphological traits are highly sensitive to progressive water deficit, as previously reported for *Populus* hybrids (Marron et al. 2003, Monclus et al. 2006). In the case of ED, the impairments were particularly severe. All measured leaf traits could not be determined due to complete leaf senescence, highlighting that young *P. nigra* seedlings have limited tolerance to prolonged water deficit. Stem growth and biomass were also drastically reduced, with many ED plants failing to produce new growth even after an extended rewatering period. These observations emphasize that extreme water stress can overwhelm both physiological and morphological compensatory mechanisms, leading to irreversible damage. This catastrophic response mirrors the findings of Marron et al. (2003), who found that successive severe droughts led to irreversible reductions in growth and photosynthetic capacity in *Populus × canadensis* clones. Similarly, studies on *P. nigra* seedlings highlight that early developmental stages are particularly vulnerable to extreme water deficits due to shallow root systems, limited carbon reserves and immature stress-defense mechanisms (Monclus et al. 2006, Cocozza et al. 2010). Our results support the view that water availability is the dominant driver of tree functional responses. Grossiord et al. (2017) demonstrated that, across semi-arid ecosystems, precipitation, not temperature, is the primary determinant of carbon uptake and physiological functioning, consistent with our observation that drought severity explained most of the variance in seedling performance.

When HT was applied under OW conditions, young *P. nigra* seedlings already exhibited marked physiological and, to a lesser extent, morphological adjustments, demonstrating that heat stress alone constitutes a significant constraint even in the absence of soil water limitation. Well-watered plants grown at HTs consistently showed lower RWC and more negative LWP than OT controls, along with higher LST. Under well-watered conditions, HT increased transpiration rates and, at certain time points, stomatal conductance, indicating that elevated atmospheric demand can enhance water loss even when soil moisture is not limiting. Thus, the rise in LST reflects not only the limited effectiveness of transpirational cooling despite increased water loss, but also the combined influence of elevated VPD and stomatal regulation, which together prevent the plant from fully offsetting heat-induced leaf warming (Wahid et al. 2007, Urban et al. 2017). From a physiological perspective, HT significantly reduced Pn across most sampling dates, despite maintaining soil water availability. This reduction was accompanied by a higher transpiration rate (Tr) and lower intrinsic photosynthetic WUE, suggesting that increased stomatal opening under heat was insufficient to offset thermal limitations on carbon assimilation, likely linked to temperature-induced impairment of Rubisco activase activity and reduced stability of thylakoid membranes (Crafts-Brandner and Salvucci 2000, Hüve et al. 2011). Consistently, LCC, Fv/Fm and ΦPSII were all reduced under HT, while NPQ was enhanced, indicating a shift toward photoprotective energy dissipation to prevent overexcitation and thermal damage of the photosynthetic apparatus (Wahid et al. 2007, Murchie and Lawson 2013). The higher accumulation of superoxide anion radicals observed at several timepoints further supports the occurrence of heat-induced oxidative stress, even under non-limiting water conditions, as previously reported for poplar and other tree species exposed to elevated temperatures (Mittler 2006, Suzuki and Mittler 2006). Morphologically, heat alone promoted a decoupling between growth components: SH was consistently greater under HT, reflecting enhanced stem elongation, whereas LA, LB and SB showed transient stimulation early in the experiment, followed by stagnation and decline at later stages. Similar responses have been described in *Populus* species, where elevated temperature favored elongation growth but constrained carbon balance and biomass accumulation over time (Silim et al. 2010, Way and Oren 2010). This pattern suggests a heat-driven reallocation toward vertical growth at the expense of structural biomass, potentially reflecting a stress escape rather than a productivity-oriented strategy. Overall, these results demonstrate that elevated temperature alone induces a coordinated suite of physiological downregulation, photoprotective responses, oxidative stress and altered growth trajectories in well-watered *P. nigra* seedlings, underscoring that heat stress can substantially limit carbon gain and functional performance independently of drought.

Taken together, these results reinforce the notion that young *P. nigra* seedlings are highly susceptible to both drought and heat, with physiological traits such as water potential and photosynthetic efficiency declining in concert with morphological impairments, including reductions in LA and stem growth. Moreover, the severe and often irreversible effects of ED highlight a critical threshold beyond which seedlings cannot recover, underscoring the importance of accounting for early-stage vulnerability in forest restoration and climate adaptation strategies.

### Synergistic effects of combined drought and heat stress

Consistent with our second hypothesis, the combination of drought and HT produced synergistic effects that exceeded the magnitude of responses observed under either stress applied alone. Combined stress conditions led to more pronounced declines in photosynthetic performance, water relations and growth than drought or heat alone, suggesting that heat amplifies water-deficit limitations on both carbon assimilation and hydraulic function. Under concurrent drought and heat stress, stomatal conductance (Gs) and transpiration (Tr) become strongly constrained by drought-induced stomatal closure, while HTs elevate evaporative demand and leaf temperature. As a result, the normal coupling between stomatal opening and evaporative cooling breaks down: even though atmospheric demand for evaporation increases, stomatal closure prevents sufficient transpiration to dissipate heat (Li et al. 2025). This mismatch leads to greater leaf heating, intensifying thermal stress and accelerating photoinhibition and oxidative damage. These data are consistent with observations in other plant species seedlings subjected to heat combined with drought conditions (Raja et al. 2020). In forest trees, Rennenberg et al. (2006) emphasized that heat-drought interactions often impair both photosynthetic and respiratory processes, mirroring the declines in Pn, Fv/Fm and ΦPSII that we observed under combined stress and consistent with broader patterns where drought and heat suppress photosynthesis, alter carbon allocation and amplify oxidative stress responses. Comparable patterns have been observed in other temperate tree species. Arend et al. (2013) showed that elevated daytime temperatures enhanced photosynthesis in European oaks under well-watered conditions but that drought strongly reduced Pn, Gs and chlorophyll content and these drought effects were exacerbated when drought and elevated temperature occurred simultaneously. Furthermore, Didion-Gency et al. (2022) found that while warming alone enhanced photosynthetic properties in oak, soil moisture reduction markedly decreased photosynthetic performance in both beech and oak, and combined warm–dry conditions further reduced photosynthetic capacity, highlighting the sensitivity of tree carbon uptake to simultaneous drought and warming.

The interactive nature of drought and heat stress is also reflected in their impacts on plant water status and oxidative metabolism. In combined stress scenarios, RWC and LWP decline more rapidly, leading to early onset of cellular dehydration and intensified production of reactive oxygen species (ROS), which can overwhelm antioxidant defenses and damage membranes, proteins and photosynthetic machinery (Alhaithloul 2019). This oxidative imbalance not only suppresses carbon assimilation but also accelerates senescence, contributing to reduced growth and biomass accumulation beyond the effects observed under single stresses (Suzuki et al. 2014). In our scenario, HT ED plants were particularly vulnerable, with only half of the seedlings surviving this treatment.

Morphological traits further illustrate synergistic effects. Combined stress often leads to greater reductions in LA, height and biomass than either drought or heat alone, indicating that the co-occurrence of HT with water deficit places seedlings in a survival-oriented state where growth processes are strongly suppressed in favor of maintenance and stress avoidance. These patterns align with findings in other tree species, where the combined impact of drought and heat is typically synergistic rather than additive, leading to disproportionately stronger declines in physiological performance, carbon assimilation and survival (Burgess 2024, Field et al. 2024). Collectively, these responses reflect a complex, integrated phenotypic reprogramming under combined stress, in which the interplay among stomatal limitations, thermal disruption and oxidative damage drives plants toward stress tolerance at the expense of growth and productivity. This synergy has profound implications under future climate scenarios, in which drought and heat waves are expected to co-occur more frequently and intensively, challenging seedling establishment and forest resilience (Zscheischler et al. 2018).

### Recovery capacity depends on stress severity and temperature

In line with our third hypothesis, post-stress recovery in *P. nigra* seedlings strongly depended on both the severity of drought and the thermal context in which stress occurred. After an MD, seedlings largely recovered leaf water status and photosynthetic activity, indicating that hydraulic and metabolic functions were only transiently impaired. This is consistent with previous evidence that mild water deficits mainly impose reversible stomatal limitations on photosynthesis (Flexas and Medrano 2002, Chaves et al. 2009). In contrast, severe drought led to only partial recovery, particularly under HT conditions, suggesting the onset of non-stomatal limitations, including damage to the photosynthetic apparatus and reduced carbon assimilation capacity (Marron et al. 2003). Extreme drought (ED) led to incomplete or failed recovery, with many HT seedlings exhibiting persistent physiological and morphological impairments or mortality, likely due to irreversible hydraulic failure, carbon reserve depletion and cumulative oxidative damage (Mittler 2006, Salmon et al. 2018). Moreover, recovery of physiological traits generally preceded morphological restoration, indicating that growth processes require sustained carbon availability beyond initial functional recovery (Peters et al. 2018). Our results demonstrate that increasing drought intensity progressively constrains recovery potential and that elevated temperature further amplifies post-stress limitations, supporting the hypothesis that stress severity and thermal conditions critically determine resilience in young *P. nigra* seedlings.

### Multivariate trait coordination shifts under increasing stress

The PCA analyses support the fourth hypothesis that trait coordination shifts from growth- and photosynthesis-oriented strategies under mild stress to survival- and protection-focused strategies under severe and extreme stress. During MD, PC1 primarily captured photosynthetic performance, while PC2 reflected growth traits, indicating an active balance between productivity and stress mitigation. Under SD and ED, photosynthetic and hydraulic traits dominated the primary axes, with elevated leaf temperatures, NPQ and oxidative stress clustering together, reflecting a shift toward photoprotection and survival. During rewatering, recovery component analyses revealed incomplete re-expansion of the functional trait space, particularly under HT conditions, with physiological and structural traits failing to return to pre-stress coordination. This outcome illustrates the stress-history dependence of trait integration and highlights how combined heat and drought push seedlings toward conservative strategies prioritizing survival over growth, consistent with theory on plant stress acclimation and adaptive plasticity (Flexas et al. 2004, Tardieu et al. 2018).

## Conclusions

This study demonstrates that young *P. nigra* seedlings exhibit strong phenotypic plasticity in response to drought and heat stress, but their resilience is tightly constrained by stress severity and thermal context. Both stressors individually impaired plant water relations, photosynthetic performance and growth, while their combination produced synergistic effects that amplified physiological dysfunction, oxidative stress and growth suppression. In line with our hypotheses, post-stress recovery depended on the intensity and duration of drought and was further limited under HT conditions: MD allowed near-complete functional recovery, severe drought resulted in partial recovery and ED often caused irreversible damage, especially when combined with heat. Physiological traits generally recovered faster than morphological traits; this pattern is consistent with the well-established principle that physiological traits are inherently more plastic and responsive to short-term environmental change than morphological traits, which typically integrate stress over longer timescales and thus recover more slowly. Multivariate analyses further revealed a stress-driven shift from growth-oriented to survival-oriented trait coordination, with incomplete reorganization during recovery under HT. Overall, our findings highlight the vulnerability of early developmental stages of *P. nigra* to compound climate extremes and emphasize that future increases in drought–heat co-occurrence may severely constrain seedling establishment, forest regeneration and ecosystem resilience. Future studies should explicitly assess belowground processes and root functional traits, enabling a more complete understanding of the mechanisms underlying whole-plant functioning under compound heat and drought stress.

## Supplementary Material

Supplementary_material_tpag078

## Data Availability

Experimental data and materials will be made available to third-party academic researchers upon request to the corresponding author.
