# Supplementary material for: Combined effects of drought intensity and heat stress impair physiological performance and recovery capacity in Populus nigra L. seedlings
Source: Tree Physiol. 2026 Jun 11;46(7):tpag078. doi: 10.1093/treephys/tpag078 (PMC13358871; doi:10.1093/treephys/tpag078)
Supplement: Supplementary_material_tpag078 [file supplementary_material_tpag078.docx]

| Supplementary Table 1. Two-way aligned ranks transformation ANOVA to test the effects of drought (D: moderate, severe, extreme), temperature (T: optimal, high), and their interaction (D x T) in the drought phase, and the effects of drought, temperature, and their interaction in the rewatering phase for relative water content (RWC), leaf water potential (LWP), leaf surface temperature (LST), net photosynthetic rate (Pn), transpiration rate (Tr), stomatal conductance (Gs), water use efficiency (WUE), leaf chlorophyll content (LCC), maximum quantum efficiency of photosystem II (Fv/Fm), quantum yield of photosystem II (ФPSII), non-photochemical quenching (NPQ), superoxide anion radicals (O2-) accumulation, leaf area (LA), leaf biomass (LB), stem biomass (SB), stem height (SH), stem diameter (SD), aboveground biomass (AB), soil temperature (ST). | | | | | | | | | |
| --- | --- | --- | --- | --- | --- | --- | --- | --- | --- |
|  |  | Drought | | |  |  | Rewatering | | |
|  |  | D | T | D x T |  |  | D | T | D x T |
|  |  |  |  |  |  |  |  |  |  |
| RWC | F | 76,3 | 105,8 | 6,3 |  | F | 48,2 | 257,4 | 42,7 |
|  | *p* | **0.000** | **0.000** | **0.003** |  | *p* | **0.000** | **0.000** | **0.000** |
|  |  | *** | *** | ** |  |  | *** | *** | *** |
| LWP | F | 27,4 | 56,9 | 2,3 |  | F | 3,7 | 45,0 | 8,8 |
|  | *p* | **0.000** | **0.000** | 0.109 |  | *p* | **0.015** | **0.000** | **0.000** |
|  |  | *** | *** | ns |  |  | * | *** | *** |
| LST | F | 71,4 | 171,7 | 155,8 |  | F | 22,9 | 219,4 | 172,0 |
|  | *p* | **0.000** | **0.000** | **0.000** |  | *p* | **0.000** | **0.000** | **0.000** |
|  |  | *** | *** | *** |  |  | *** | *** | *** |
| Pn | F | 21,9 | 75,6 | 3,5 |  | F | 1,6 | 95,3 | 6,6 |
|  | *p* | **0.006** | **0.000** | **0.036** |  | *p* | 0.197 | **0.000** | **0.000** |
|  |  | ** | *** | * |  |  | ns | *** | *** |
| Tr | F | 24,1 | 168,1 | 66,0 |  | F | 0,8 | 127,4 | 9,4 |
|  | *p* | **0.005** | **0.000** | **0.000** |  | *p* | 0.498 | **0.000** | **0.000** |
|  |  | ** | *** | *** |  |  | ns | *** | *** |
| Gs | F | 26,7 | 26,4 | 8,8 |  | F | 3,6 | 8,6 | 3,0 |
|  | *p* | **0.004** | **0.022** | **0.000** |  | *p* | **0.017** | **0.004** | **0.035** |
|  |  | ** | * | *** |  |  | * | ** | * |
| WUE | F | 1,0 | 63,3 | 2,2 |  | F | 1,5 | 176,1 | 1,9 |
|  | *p* | 0.394 | **0.000** | 0.122 |  | *p* | 0.224 | **0.000** | 0.138 |
|  |  | ns | *** | ns |  |  | ns | *** |  |
| LCC | F | 88,7 | 196,6 | 60,9 |  | F | 26,2 | 205,4 | 83,2 |
|  | *p* | **0.000** | **0.000** | **0.000** |  | *p* | **0.000** | **0.000** | **0.000** |
|  |  | *** | *** | *** |  |  | *** | *** | *** |
| Fv/Fm | F | 19,9 | 131,9 | 6,1 |  | F | 14,2 | 13,2 | 2,6 |
|  | *p* | **0.004** | **0.000** | **0.003** |  | *p* | **0.000** | **0.000** | **0.059** |
|  |  | ** | *** | ** |  |  | *** | *** | . |
| ФPSII | F | 8,7 | 177,0 | 4,1 |  | F | 5,3 | 107,5 | 0,3 |
|  | *p* | **0.000** | **0.000** | **0.021** |  | *p* | 0.051 | **0.000** | 0.845 |
|  |  | *** | *** | * |  |  | . | *** | ns |
| NPQ | F | 24,8 | 175,6 | 72,7 |  | F | 95,7 | 250,6 | 42,4 |
|  | *p* | **0.001** | **0.000** | **0.000** |  | *p* | **0.000** | **0.000** | **0.000** |
|  |  | *** | *** | *** |  |  | *** | *** | *** |
| O_2_^-^ | F | 4,5 | 38,1 | 23,8 |  | F | 3,7 | 1,7 | 11,8 |
|  | *p* | **0.014** | **0.000** | **0.000** |  | *p* | **0.014** | 0.190 | **0.014** |
|  |  | * | *** | *** |  |  | * | ns | * |
| LA | F | 22,0 | 0,0 | 3,1 |  | F | 26,7 | 7,3 | 10,0 |
|  | *p* | **0.000** | 0.902 | 0.053 |  | *p* | **0.000** | **0.009** | **0.000** |
|  |  | *** | ns | . |  |  | *** | ** | *** |
| LB | F | 28,5 | 3,0 | 4,1 |  | F | 24,2 | 0,1 | 5,4 |
|  | *p* | **0.000** | **0.087** | **0.020** |  | *p* | **0.000** | 0.708 | **0.002** |
|  |  | *** | . | * |  |  | *** | ns | ** |
| SB | F | 21,7 | 3,4 | 6,6 |  | F | 9,9 | 8,0 | 13,5 |
|  | *p* | **0.000** | 0.070 | **0.000** |  | *p* | **0.000** | **0.006** | **0.003** |
|  |  | *** | . | *** |  |  | *** | ** | ** |
| SH | F | 26,0 | 43,3 | 9,2 |  | F | 11,0 | 58,8 | 2,2 |
|  | *p* | **0.000** | **0.000** | 0.238 |  | *p* | **0.002** | **0.000** | 0.093 |
|  |  | *** | *** | ns |  |  | ** | *** | . |
| SD | F | 35,3 | 0,6 | 6,5 |  | F | 8,7 | 20,0 | 0,4 |
|  | *p* | **0.000** | 0.438 | **0.001** |  | *p* | **0.000** | **0.000** | 0.774 |
|  |  | *** | ns | *** |  |  | *** | *** | ns |
| AB | F | 164,2 | 2,1 | 9,7 |  | F | 28,0 | 0,2 | 6,9 |
|  | *p* | **0.000** | 0.150 | 0.141 |  | *p* | **0.000** | 0.693 | **0.000** |
|  |  | *** | ns | ns |  |  | *** | ns | *** |
| ST | F | 37,4 | 327,0 | 47,8 |  | F | 2,2 | 252,0 | 48,8 |
|  | *p* | **0.000** | **0.000** | **0.000** |  | *p* | 0.092 | **0.000** | **0.000** |
|  |  | *** | *** | *** |  |  | . | *** | *** |

*** *p* < 0.001, ** *p* < 0.01, * *p* < 0.05, . *p* < 0.1, ns *p* > 0.1. Bold values indicate statistical significance (*p* < 0.05).
